# Differential Range Use between Age Classes of Southern African Bearded Vultures *Gypaetus barbatus*

**DOI:** 10.1371/journal.pone.0114920

**Published:** 2014-12-31

**Authors:** Sonja Krüger, Timothy Reid, Arjun Amar

**Affiliations:** 1 Percy FitzPatrick Institute of African Ornithology, DST/NRF Centre of Excellence, University of Cape Town, Rondebosch, South Africa; 2 Ezemvelo KZN Wildlife, Cascades, South Africa; Cornell University, United States of America

## Abstract

Bearded Vulture *Gypaetus barbatus* movements were investigated in southern Africa to determine whether an individual's age, sex or breeding status influenced its ranging behaviour and to provide the information required to guide conservation activities. Data from satellite transmitters fitted to 18 individuals of four age classes were used to determine range size and use. Because of the nature of the movements of marked individuals, these data could be used to determine the overall foraging range of the entire population, which was estimated to be 51 767 km^2^. Although juvenile, immature and sub-adult birds used different parts of the overall range, their combined foraging range was 65% (33 636 km^2^) of the overall range. Average adult home ranges (286 km^2^) were only around 1% the size of the average foraging ranges of non-adults (10 540 –25 985 km^2^), with those of breeding adults being even smaller (95 km^2^). Home ranges of breeding adults did not vary in size between seasons but adults utilized their home range more intensively whilst breeding, moving greater distances during the incubation and chick hatching period. Range size and use increased as non-adults aged. Immatures and sub-adults had larger range sizes during winter, but range use of non-adults did not vary seasonally. Range size and use did not differ between the sexes in any of the age classes. Information on home range size and use enables specific areas within the species' range to be targeted for management planning, education and conservation action.

## Introduction

As human populations increase and infrastructure such as houses, roads and power lines expands into previously undeveloped areas, many species which were previously shielded from anthropogenic influences are exposed to the impacts of human development and their associated threats [1]–[3]. Mitigating any such threats requires a clear understanding of how species use their environment in both space and time, and such knowledge can play a critical role in designing effective conservation management strategies [4]–[7].

The spatial and temporal use of the environment may differ according to an individual's age, sex or breeding status and knowledge of these differences may further contribute to ensuring that management actions are targeted appropriately [7]–[8]. This may be particularly important for species which take a long time to mature [9]–[11]. For example, large raptors often do not secure territories until they are several years old and their exploratory behaviour during this period may expose them to multiple threats across the wider landscape [12]–[15]. Non-adult birds may therefore be exposed to different threats or different levels of threat than those found for adults [11], [16]–[18]. In large raptors, non-adults form a large proportion of the population [12], [19]–[20], thus conservation measures designed to protect breeding birds only may not be sufficient to safeguard the population as a whole [8], [21].

The Bearded Vulture *Gypaetus barbatus* is a large scavenging, sexually monomorphic [22] raptor that nests on high mountain cliffs in Africa, Europe and Asia and forages extensively over the surrounding mountains [23]–[24]. Declines in Bearded Vulture populations have been documented throughout their range [23], [25]–[30], resulting from threats such as habitat loss, reduced food availability, poisoning, direct persecution, and fatal collisions with energy infrastructure [31]–[32]. In southern Africa the species is restricted to the Maloti-Drakensberg Mountains of Lesotho and South Africa, where its range and population size have declined markedly in the last few decades and there are now no more than 110 currently occupied territories [33]–[34]. The primary risks to this population are the use of poisons and the risk of collisions with energy structures (e.g., power lines and wind turbines) [13], [33], [35]–[36]. The southern African population is classified regionally as “Critically Endangered” as a result of these declines and the on-going threats faced by the population throughout its foraging range [37].

A South African Biodiversity Management Plan, ratified by government, has been developed for the species [38]. The primary objective of this plan is to halt the population decline in the short term. For the successful implementation of this plan, it is recognized that improved knowledge is required on how the species uses its environment. The size of the species' range in southern Africa and the political boundaries it encompasses make the planning, resourcing, coordinating, implementing and monitoring of conservation actions challenging. Therefore, detailed information on the spatial and temporal movements of Bearded Vulture will enable the prioritization of specific age classes or areas for focused action and the recommendation of suitable mitigatory measures for proposed developments, such as the mitigation of unsafe energy infrastructure within high use areas of their home range.

The persistence of a threatened species relies on its breeding population and the protection of their breeding territories, particularly during the breeding season [12], [39]. In order to prioritize the breeding segment of the population for protection, information on breeding territory size is required. Previous studies have shown that breeding Bearded Vultures defend a territory around their nest site and that the size of their foraging range varies throughout the year [40]–[43]. However, the core home range size for the southern African population remains unknown. Non-breeding birds on the other hand do not defend a territory and lead a nomadic lifestyle prior to breeding [42], [44]–[45]. They are thought to inhabit more marginal areas within the species' range in terms of resource availability and higher human impacts and therefore, in theory, may be exposed to a higher number and a wider range of threats throughout the year which may negatively affect their survival prospects [40].

Information on the ranging behaviour of Bearded Vulture in southern Africa is limited to five individuals tracked using conventional VHF radio-telemetry in the 1980s [40] and a single juvenile tracked for less than 10 months using GPS satellite telemetry [45]. This study therefore aims to provide a comprehensive understanding of the ranging behaviour of this population using a larger sample size across all age classes tracked over a number of years/seasons with highly accurate GPS satellite technology which provides frequent and accurate locational data.

We studied the movements of 18 Bearded Vultures in southern Africa between 2007 and 2014 and describe where (i.e., home range size) and how far (i.e., distances moved between points) individuals range and whether these parameters differ with age, sex and season/breeding status. Based on our findings on how Bearded Vultures utilize their environment, we delineate areas for the implementation of actions in the Biodiversity Management Plan for the species to guide conservation agencies in the effective use of resources. Additionally in this paper we describe whether any particular age class is more exposed to anthropogenic influences as a result of their ranging behaviour and discuss the conservation implications of our findings.

## Materials and Methods

### Study Area

**Figure 1:**
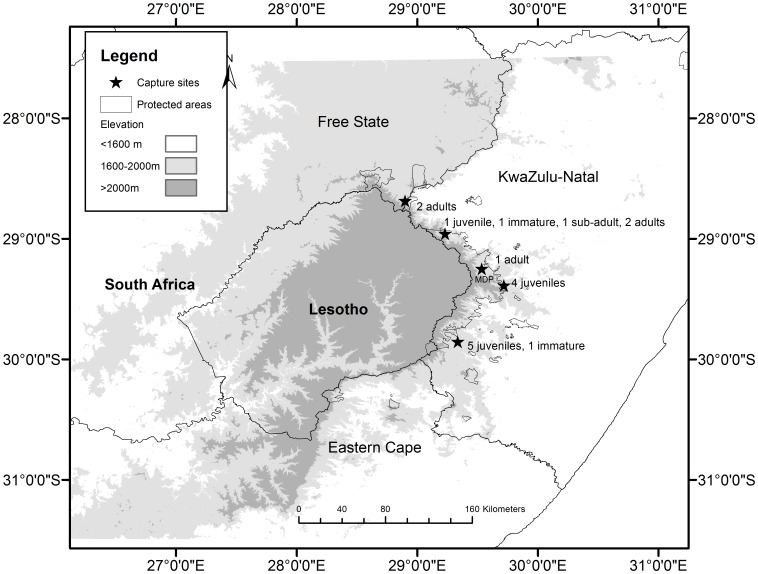
Study area and capture sites. The location of the capture sites and protected areas (the largest being the Maloti Drakensberg Park, MDP) within the Bearded Vulture's distribution range in the Maloti-Drakensberg region of Lesotho and South Africa, where the darker shades indicate higher altitudes.

The study was undertaken in the Maloti-Drakensberg region of southern Africa which spans the highlands and Maloti Mountains of Lesotho and the Drakensberg mountain range of the Free State, KwaZulu-Natal and Eastern Cape provinces of South Africa between 28°0′0″–32°0′0″ S and 27°0′0″–30°0′0″ E (Fig. 1). There is great variation in the topography of the Maloti-Drakensberg mountains with summit plateaux and peaks, vast basalt and sandstone cliffs, deep valleys and intervening spurs with an average altitude of 2 200 m (1 280 m–3 500 m) [46]. The Maloti Drakensberg Park (MDP), an inland mountain protected area totalling 242 813 ha, forms a large portion of the international boundary between KwaZulu-Natal and Lesotho. The land use in the remaining study area is predominantly commercial and communal farmland in South Africa and communal rangeland in Lesotho which is extensively grazed by livestock.

The study area encompasses the entire distribution range of the Bearded Vulture population in southern Africa, estimated at 352–390 individuals [34].

### Capture and Marking

Eighteen Bearded Vultures were fitted with satellite Platform Transmitter Terminals (PTTs) between September 2007 and September 2012 in the Maloti-Drakensberg region; 10 juveniles, two immatures and six adults (Fig. 1). Birds were caught at supplementary feeding sites (vulture restaurants) using a noose carpet with pieces of meat, fat and bone as bait, and fitted with 70 g solar-powered GPS-PTT-100s (Microwave Telemetry Inc., Maryland, USA). The individuals were aged according to criteria in [47]; juvenile (post-fledging to 2 years), immature (2–4 years), sub-adult (4–6 years) and adult (>6 years). Where possible, ≤2 ml of blood was taken per individual for genetic and heavy metal analysis as well as sex determination, using the sexing kit supplied by Molecular Diagnostic Services (MDS Pty Ltd., Westville, South Africa) for sample collection. Genetic sex determination was performed by MDS using nucleic acid amplification procedures with blood taken from the brachial vein (n = 14) or from the tip of the feather shaft of a breast feather (n = 4) if it was not possible to take blood.

In all cases, PTTs were attached using a pelvic harness attachment [48]. The harnesses were constructed using a 2 mm silicon cord inserted into 0.25″ tubular Teflon which for added strength was then inserted into 0.33″ tubular Teflon Ribbon (Bally Ribbon Mills, Bally, Pennsylvania). The harnesses were constructed using a weak link sewn with dental floss initially (n = 15), but after these proved too durable they were replaced by cotton thread (n = 6) to allow birds to lose the harness after the end of the PTT′s life cycle [49], predicted to be between five to eight years.

The PTTs recorded one GPS position per hour from 05∶00 to 20∶00 hours (local time) daily as well as date, time and the instantaneous speed at the time of the recorded position, either in kilometers per hour (older PTTs) or knots (newer PTTs).

### Ethics Statement

Vulture capture and marking procedures were approved by the Animal Ethics Committee of the Science Faculty of the University of Cape Town (reference: 2001\V14\SK), South African National Parks and Ezemvelo KwaZulu-Natal Wildlife (Research Project Registration number W/2057/01). Capture and handling of vultures and the fitting of tracking units were executed under the Endangered Wildlife Trust's Threatened or Protected Species registration certificate granted by the Gauteng Provincial Department of Agriculture, Conservation and Environment, South Africa (permit: 07046).

### Spatial and Temporal Analyses

For all spatial analyses the GPS fixes were projected to the UTM coordinate system (WGS 1984 UTM Zone 35S) for use in R v.3.0.2 [50], ArcGIS v.10.0 (ESRI, Redlands, USA) and the Geospatial Modelling Environment (GME) [51]. For all temporal analyses we compared two seasons. For the non-adult age classes, our “season” variable was either summer (1 October–31 March) or winter (1 April–30 September), based on the number of daylight hours, because we expected home range size and use to vary according to food availability which is known to vary seasonally. For adults, “season” was either breeding (1 May–31 December) or non-breeding (1 January–30 April) because we expected home range size and use to vary with the type of breeding activity. We defined the breeding season as the period between courtship and nest building until fledging, and the non-breeding season as the post fledging period until natal dispersal upon initiation of nest building the following year [52]. For all spatial analyses involving either monthly or seasonal comparisons, we used only data from individuals that were tracked for at least an entire month or an entire season respectively. Means are presented as mean ± standard deviation throughout.

### Home Range Size

The home range or utilization distribution of each individual was estimated by means of a kernel density approach [53]–[55]. Total and monthly home range sizes were calculated in R using the package “adehabitatHR” v.0.4.10 [56] with the package “rgdal” v.0.8-16 [57] to process the spatial data.

Home range estimates were derived by drawing contour lines (i.e., isopleths) based on the volume of the curve under the utilization distribution [58] which defined home range polygons whose areas were then calculated. These were estimated using a kernel function [59]–[60]. The utilization distribution was estimated using a bivariate normal kernel function so that the probability density of the locations of the individuals followed the XY coordinates [60]. Fixed 90%, 75% and 50% kernel density contours were calculated to estimate the majority of the home range areas (90%), and the core (intensive use) areas (50%) [61]. The smoothing parameter was computed with the *ad hoc* method [56]. The utilization distribution was estimated over a grid of a smaller size for adults (100 spatial pixels) than for non-adults (450 spatial pixels) because adult GPS fixes were concentrated in a much smaller area.

Additionally we merged the 90%, 75% and 50% kernel ranges of the three non-adult age classes to determine the geographical and administrative areas that were overlapped by each of these kernels for management planning purposes, i.e. the intensively used area (50% kernel) represents the minimum area for the implementation of conservation action.

We also calculated the overall foraging range of each individual as the Minimum Convex Polygon (MCP) encompassing all GPS fixes obtained for that individual [54]. Although MCPs have a tendency to overestimate the actual area occupied by the individual [62], they provide an indication of the overall foraging area and allow comparisons with historical studies.

### Home Range Use

To quantify the extent of vulture movements, we determined the distance between hourly fixes for individuals across all age classes. Using GME, hourly flight distances were calculated as the straight-line distance between consecutive fixes that were separated by one hour within the same day, providing a minimum hourly distance travelled. For the analyses we used mean hourly distances per month for each individual.

### Statistical Analyses

See S1 Table (in the Supporting Information) for a summary of the analyses described below. We used the “lme4” v.1.0-6 package [63] within R to perform Linear Mixed Models (LMM) with Wald chi-square tests to explore the relationships between age and i) home range size (S1 Table, I), and ii) hourly flight distances (i.e., home range use) (S1 Table, II).

We calculated total range sizes and mean hourly distances travelled per month for each age class as described above. We then compared these between age classes and sexes by fitting age, sex and the interaction between them as fixed factors in the model (S1 Table). The interaction between sex and age explored whether any difference in home range size or use between the sexes was consistent for each age class. Because duration of tracking may influence the accuracy of home range size, we included the log of the number of months each bird was tracked as a weighting term in the analyses comparing range size between age classes. In doing so we therefore attempted to account for the variable length of time for which we tracked different birds. Month and year were included as fixed factors in the home range use model to control for the effect of month or year on distances moved, because data were not fully balanced between these variables for each individual. Individual identity was included as a random term in these LMMs to account for the lack of independence between individuals in the different age classes because some birds crossed age classes as they aged. Pairwise comparisons between age classes and between the interaction terms were made using the “lsmeans” v.1.10-4 package [64] with *P* values adjusted using the Tukey method [65], the default for pairwise comparisons among adjusted means.

We then compared adult home range size and use between seasons using the home range size/use per breeding individual for each season as the response variable and fitting season and year as fixed factors in the model. Individual identity was again included as a random term because we had multiple years of data from some individuals. Some adults failed to breed (n = 2) and the breeding season data from these non-breeding individuals were excluded for seasonal and monthly (see below) comparisons. We used this same model structure to examine temporal patterns of range size and use for non-adults (juveniles, immatures and sub-adults). For these seasonal analyses we grouped age classes where appropriate (see results).

To investigate temporal patterns of home range size and use by breeding adults throughout the year at a finer scale, we repeated these same models but with month, sex and year as fixed factors and monthly home range size per individual as the response variable. Although the sexes did not differ in total home range size (see results), we included sex as a factor in our monthly and seasonal comparisons, and the interaction between sex and month, and sex and season to explore whether breeding male and female home range sizes differed between months and seasons.

## Results

### Home Range Size

We obtained satellite tracking data from 18 Bearded Vultures tracked for 392 bird-months (146 607 GPS fixes) between September 2007 and April 2014; 85 months of juvenile movements (22%), 113 months of immature movements (29%), 31 months of sub-adult movements (8%) and 163 months of adult movements (41%). For full details of each individual tracked see S2 Table.

**Figure 2:**
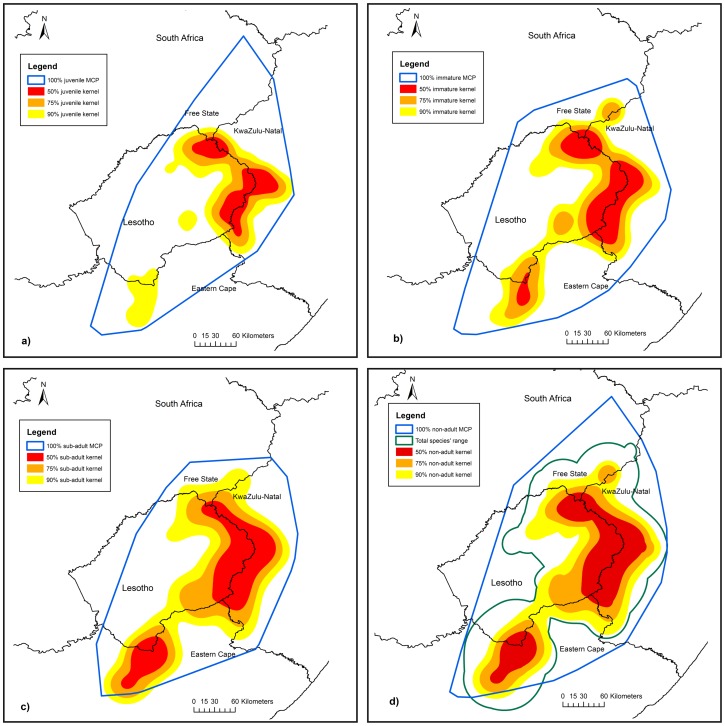
Geographic location of non-adult home ranges. Bearded Vulture Minimum Convex Polygons (MCP) and 50%, 75% and 90% kernel home ranges in southern Africa showing the total range collectively for a) juveniles (n = 10), b) immatures (n = 7), c) sub-adults (n = 3), and d) the merged ranges of non-adults (n = 20), shown in relation to the overall range for the species indicating the geographic area in which to focus conservation action outside of protected areas.

**Figure 3:**
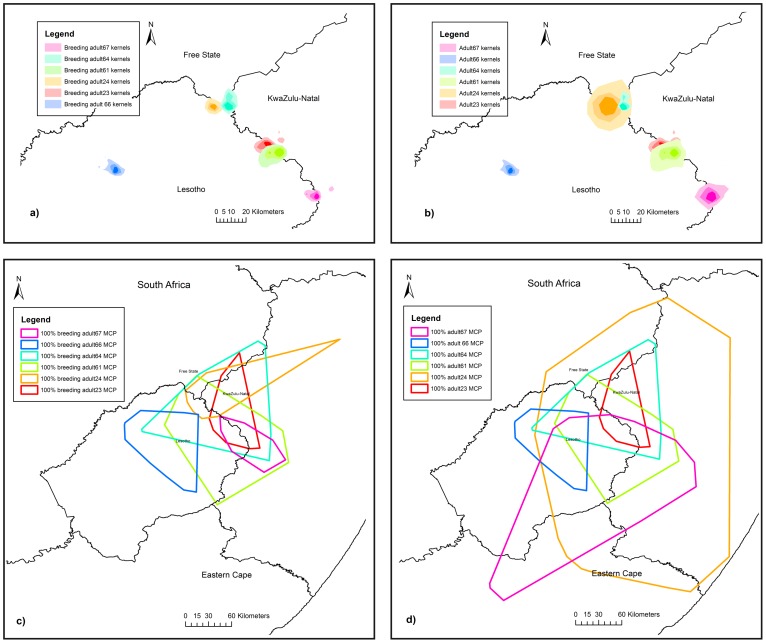
Geographic location of adult home ranges. Bearded Vulture home ranges in southern Africa showing 50%, 75% and 90% kernel home ranges for a) breeding adults (n = 6) and b) all adults (n = 6), and Minimum Convex Polygon (MCP) home ranges for c) breeding adults (n = 6) and d) all adults (n = 6), indicating some overlap of home ranges.

The total area of use of all age classes was 51 767 km^2^ based on the 90% kernels of all individuals combined. The kernel and MCP ranges of marked birds in this study covered the documented range for the species [66] and can therefore be considered the foraging range of the entire population. The foraging areas of non-breeding birds (n = 12) covered most of this area (Fig. 2) whereas adult (n = 6) home range areas were focused around their specific breeding territories with some overlap between territories (Fig. 3) and large variation between individual MCPs, particularly in the non-breeding season (Fig. 3d). The merged 50%, 75% and 90% kernels of non-adults, covered an area of 10 982 km^2^, 21 454 km^2^ and 33 636 km^2^ respectively, of which the 90% kernel covered 65% of the populations' foraging range (Fig. 2d).

There was a significant difference in overall range size between age classes (χ^2^_(3)_ = 63.99, P<0.01) with pairwise comparisons showing that the ranges of all age classes differed significantly in size (P<0.01) apart from immature and sub-adult ranges (P = 0.16) (Table 1). Non-adult range sizes increased as birds aged, prior to becoming adults but the home range size of adults, particularly breeding adults, was significantly smaller than those of all other age class (Table 1, S3 Table). There was no significant difference in overall range size between sexes (χ^2^_(1)_ = 0.19, P = 0.66), although the interaction between age and sex was significant (χ^2^_(3)_ = 17.88, P<0.01). Range sizes of males and females did not differ significantly within each age class but the difference in immature male (17 254±2155 km^2^) and female (26 802±2184 km^2^) range sizes was close to significant (P = 0.06) and resulted in the significance of the interaction term. Home ranges estimated by MCPs showed a similar trend of increasing range size with age, although they were much larger overall (Fig. 2, Table 1).

**Table 1:** A comparison of the total and seasonal 90% kernel home range estimates in km^2^ (mean ± standard deviation) and the Minimum Convex Polygon (MCP) home range estimates for different age classes of Bearded Vulture in southern Africa.

Age class	Total 90% kernel range size in km^2^	Total MCP range size in km^2^	Seasonal 90% kernel range in km^2^	
			Non-breeding/Summer	Breeding/Winter
Juvenile (n = 10)	10 540±7 306	21 151±9 888	9 504±5 038	10 640±5 082
Immature (n = 7)	21 880±8 187	34 188±11 081	13 167±13 999	29 836±8 362
Sub-adult (n = 3)	25 985±7077	40 961±9 274	27 272±11 005	33 573±8 497
Adult (total) (n = 6)	286±361	18 751±23 385	n/a	n/a
Adult (breeding) (n = 6)	95±19	5 220±3 850	105±62	148±108

Adult home ranges did not differ significantly between the breeding (148±62 km^2^) and non-breeding (105±108 km^2^) seasons (χ^2^_(1)_ = 3.21, P = 0.07), nor did they differ between months (χ^2^_(1)_ = 0.67, P = 0.41). There were also no differences in monthly home range size between sexes (χ^2^_(1)_ = 0.01, P = 0.94). Since the total foraging ranges of immatures and sub-adults were similar (see above), these data were combined for seasonal analyses. Juvenile foraging ranges did not differ significantly seasonally (χ^2^_(1)_  = 2.84, P = 0.09) but foraging ranges of immatures and sub-adults were on average significantly larger (χ^2^_(2)_ = 15.37, P = <0.01) during winter than in summer.

### Home range use

Monthly mean hourly distances travelled varied significantly between age classes (χ^2^_(3)_ = 11.81, P<0.01) but not between sexes within each age class (χ^2^_(1)_ = 0.26, P = 0.61). There was also no significant difference in the interaction between age and sex (χ^2^_(3)_ = 5.87, P = 0.12). Pairwise tests showed that adults travelled significantly shorter distances than immatures (P = 0.03) and sub-adults (P = 0.04) but travelled similar distances to juveniles (P = 0.07). Non-adult age classes did not differ significantly from each other in the mean distances travelled per month (P>0.05 in all cases) (Table 2), therefore these data were combined for further analyses. Range use reflected the general patterns of range size, with adults travelling the shortest distances and distances increasing in non-adults as they aged (Table 2, S3 Table).

**Table 2:** A comparison of the average hourly distances (in km) between fixes (mean ± standard deviation) of the different age classes of Bearded Vulture; sample sizes and ranges are also displayed.

Age class	Average hourly distance travelled	Non-breeding/Summer	Breeding/Winter
Juvenile (n = 10)	5.2±1.2 km (n = 7: 0–51 km)	5.0±1.2 km	5.5±1.2 km
Immature (n = 7)	6.8±1.6 km (n = 10: 0–123 km)	6.7±1.8 km	7.0±1.3 km
Sub-adult (n = 3)	7.8±1.8 km (n = 3: 0–109 km)	7.6±1.8 km	8.1±1.9 km
Adult (n = 6)	4.1±1.4 km (n = 6: 0–184 km)	3.2±0.8 km	4.4±1.5 km

Pairwise comparisons of average distances indicated significant differences only between adults and immatures (P = 0.03), and adults and sub-adults (P = 0.04). Adult movements differed significantly between seasons (P<0.001) whereas non-adults did not (P = 0.16).

**Figure 4:**
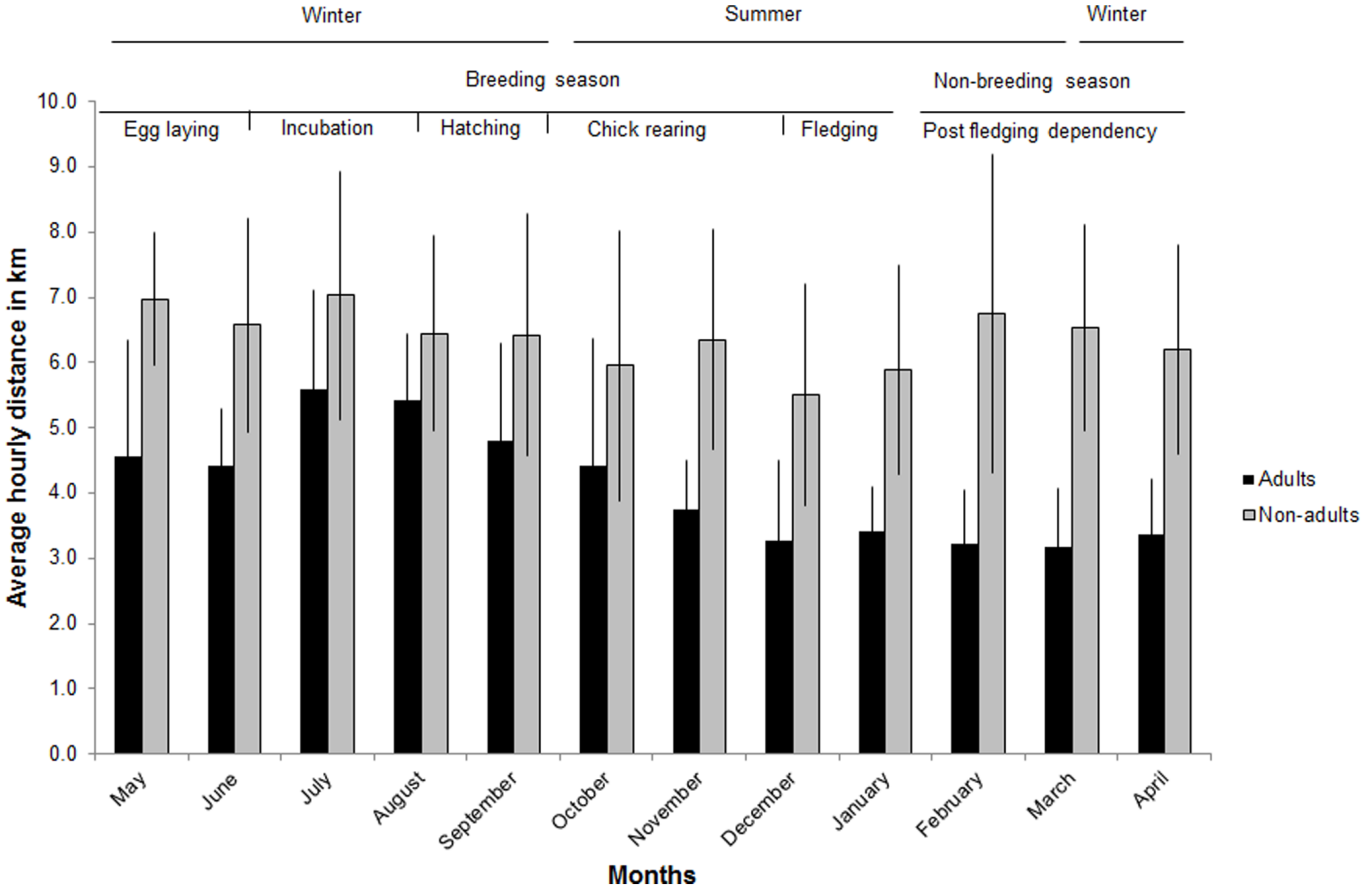
Hourly distances travelled. The mean (mean ± standard deviation) monthly distances (in km) per hour between fixes of breeding adults, showing an increase in distances moved at the start of the breeding season in May, a peak during the incubation and hatching period and a decrease during the fledging period at the end of the breeding season (December) and during the post fledging period. The average (mean ± standard deviation) monthly distances per hour (in km) of non-adults are shown for winter and summer.

The movements of breeding adults varied significantly according to season (χ^2^_(1)_ = 34.89, P<0.001) and month (χ^2^_(11)_ = 58.06, P<0.001), with birds moving further between hourly fixes during the breeding season than the non-breeding season (Table 2) particularly during incubation and chick hatching (Fig. 4). Sexes, however, did not differ in their movements either between seasons (χ^2^_(1)_ = 3.32, P = 0.07) or months (χ^2^_(1)_ = 0.02, P = 0.89) and there were also no significant interactions between sexes and seasons (χ^2^_(1)_ = 1.61, P = 0.20), and sexes and months (χ^ 2^_(1)_ = 7.98, P = 0.71). There were no significant seasonal differences in the movements of non-adults (χ^ 2^_(1)_ = 1.98, P = 0.16) (Table 2).

## Discussion

This study showed that the home range size of breeding adults was relatively small compared to that of non-breeding adults. The range in size of the 90% kernel home range areas of all adults in this study (77–1 000 km^2^) encompasses the average adult territory size recorded in the Pyrenees (300 km^2^) [67] and the Caucasus (206 km^2^) [68], although those of breeding adults in this study (73–127 km^2^) were smaller. However, the MCP home ranges calculated for breeding adults in this study (2 726–12 343 km^2^) were 10 times larger than those estimated by [40] for the same population using radio telemetry (302–743 km^2^) and those home range areas calculated from inter-nest distances (625 km^2^) [19], most probably because of the use of improved tracking technology. The variation in home ranges between breeding adults in our study may be as a result of differences in food availability whereas some non-breeding adults may be travelling large distances in search of a mate if mate loss was the reason for them not breeding in a particular year. This deserves further investigation.

The average MCP home range area of juveniles in this study (21 151 km^2^, n = 12) was almost 10 times larger than those of juveniles in the Pyrenees (2 225 km^2^, n = 3) [69]. Our MCP calculations for non-adults (21 151–40 961 km^2^) were nearly identical to earlier findings of [70] for non-adults (23 683 km^2^–40 932 km^2^) within a similar sized distributional range in the Spanish-French Pyrenees but were larger than more recent estimates from the Pyrenees (945–19 008 km^2^) [69]. Again our estimates were much larger than the immatures tracked through conventional VHF radio telemetry by [40] which were described as having three areas of use, averaging 608 km^2^ each (i.e., a total area of 1 825 km^2^). Our findings of increased range size and use with age in non-adults support the findings of [70] and [71] who suggest that range size and use increases with age as the individuals explore their territory.

Although adults maintained the same home range size throughout the year, in contrast to the findings of [40] and [70], their use of home range (based on minimum distances moved) was higher during the incubation and hatching/early chick rearing stages of the breeding cycle. Our findings suggest that breeding adults need to increase their search distances and intensity whilst breeding and spend less time on non-foraging related activities because of the need to return to the nest frequently. Incubation and chick rearing duties are shared by the sexes [52], therefore the energy demand of both birds may be high after long periods of inactivity whilst incubating or guarding the chick. Winter is also a period of food shortage in the region because livestock are moved to lower altitudes and ungulate deaths are low at the beginning of the season thus birds may be required to fly further in search of food. Our theory of increased search distances and intensity during incubating and chick hatching is supported by the tracking data, which on subsequent inspection showed that during these periods the birds spent only 22% of their time moving distances of less than 1 km per hour compared with 35% of the time in other months. In addition the frequency of movements greater than 10 km between fixes doubled during winter (13%) which is indicative of more extensive searching when food resources are scarce. Breeding adults may therefore benefit from the provision of a consistent and regular supply of food close to their nest sites to reduce the need to range more extensively in the breeding season thus reducing exposure to threats and increasing breeding success.

Non-adults on the other hand increased the size of their range in winter but not the use of their range. The idea that the increased foraging range of immatures and sub-adults during winter may also be in response to food scarcity, is supported by the findings of [44] who showed that variation in ranging behavior of non-adults was indicative of a spatially unpredictable or highly dispersed food resource. The movements may also be a response to interactions with conspecifics, territory exploration with age (immatures) and searching for a partner (sub-adults) [40], [45]. Therefore non-adults would also benefit from supplementary feeding during winter with sites located in core areas of their foraging range.

We found no differences between the sexes in either range size or use. This result is to be expected for a sexually monomorphic species [22] where parental duties are shared by adults [54]. Similarly [71] also found no dispersal differences between sexes in pre-adults and causes of mortality did not vary between sexes [30].

The average home range size of adults in this study can help guide the size of the area (10 km radius) required to be conserved around each of the 109 breeding territories identified by [34] for protection to safe-guard the breeding portion of the population. The radius of the core area (50% kernel) of use (4 km radius) represents the absolute minimum area for protection. The protection of the breeding territories will also benefit fledglings for the first few months before they disperse from their natal area. Similarly, the core area of the combined non-adult range (10 982 km^2^) also represents the minimum area to be conserved. However, since non-adult birds spread their activity over such large areas, the 75% (21 454 km^2^) and ideally the 90% kernels (33 636 km^2^) depict the areas in which resources for the implementation of actions need to be focused to effectively address the risks faced by non-adult birds.

Poisoning and collisions with power lines have been identified as the primary risks to the population [13], [33], [35]–[36] and were also the cause of death of the 10 marked birds found dead during this study (Krüger unpublished data). Actions for implementation in the core areas identified in this study to address these threats include; i) the mitigation of existing and proposed energy structures to reduce collision risks, ii) the establishment and improved management of supplementary feeding sites to reduce the risk of exposure to human persecution and poisoning incidents, and iii) focussed outreach programmes aimed at reducing poisoning incidents.

The larger sample size and the use of satellite telemetry in this study have provided more substantial information on the spatial and temporal movements of various age classes of Bearded Vulture in southern Africa. Since the movements of the marked birds in this study are restricted to the study area and are representative of the entire population, this information should greatly aid conservation management planning for this species and should enable key requirements of the Biodiversity Management Plan for the species [38] to be addressed This is particularly important for the conservation of a species inhabiting an area spanning both international and regional boundaries in a landscape where human activities place vulture populations at risk in the long term. Although other raptor studies have looked at human influences, space use and conservation implications [14], [72]–[76], these have often been limited because of their sample sizes or have they been focused on specific age classes, thus limiting the recommendations that can be made at the scale of the populations' foraging range.

We demonstrate that combining home range size and use estimates facilitates a more comprehensive understanding of where and when to address current and future threats to optimize conservation management strategies for a critically endangered population.

## Supporting Information

S1 Table **The key analyses with a description of the analytical approach used, specifying the response and explanatory terms (fixed and random terms) included in models.**
(DOCX)

S2 Table **Details of the 18 Bearded Vultures caught in southern Africa and the tracking information used for analyses between September 2007 and April 2014.**
(DOCX)

S3 Table **Bearded Vulture ranging information showing average hourly flight distances and kernel and MCP home range sizes in km^2^ depicted by individual separated by age class.**
(DOCX)

S1 Form **Data Access Form.** The satellite tracking data used in this study is available on request from Ezemvelo KwaZulu-Natal Wildlife.
(DOC)
