# Supplementary material for: Differential Range Use between Age Classes of Southern African Bearded Vultures Gypaetus barbatus
Source: PLoS One. 2014 Dec 31;9(12):e114920. doi: 10.1371/journal.pone.0114920 (PMC4281122; doi:10.1371/journal.pone.0114920)
Supplement: S1 Table — The key analyses with a description of the analytical approach used, specifying the response and explanatory terms (fixed and random terms) included in models. (DOCX) [file pone.0114920.s001.docx]

**Supporting Information**

**Table S1.** The key analyses with a description of the analytical approach used, specifying the response and explanatory terms (fixed and random terms) included in models.

| Analytical approach | | | |
| --- | --- | --- | --- |
| **I. Comparing home range sizes between individuals (Linear Mixed Models)** | | | |
|  | | | |
| Analysis | Response term | | Explanatory terms (random terms in parentheses): Distribution |
| 1a) Influence of age class and sex on total home range size | Total 90% kernel home ranges | | Age, Sex, Age*Sex, (Individual): Normal,  log (number of months of data) |
| 1b) Influence of season on home range of breeding adults | 90% kernel home ranges by season | | Season, Year, (Individual): Normal |
| 1c) Influence of month on home range of breeding adults | 90% kernel home ranges by month | | Sex, Month, Year, (Individual): Normal |
| 1d) Influence of season on home range of juveniles | 90% kernel home ranges by season | | Season, Year, (Individual): Normal |
| 1e) Influence of season on home range of immatures and sub-adults | 90% kernel home range by season | | Season, Year, (Individual): Normal |
| **II. Comparing hourly distances moved between individuals (Linear Mixed Models)** | | | |
| Analysis | Response term | Explanatory terms (random terms in parentheses): Distribution | |
| 2a) Influence of age class and sex on average hourly distances | Average hourly distance | Age, Sex, Age*Sex, Month, Year, (Individual): Normal | |
| 2b) Influence of season on distance of breeding adults | Average hourly distance by season per year | Sex, Season, Sex*Season, Year, (Individual): Normal | |
| 2c) Influence of month on distance of breeding adult | Average hourly distance by month per year | Sex, Month, Sex*Month, Year, (Individual): Normal | |
| 2d) Influence of season on distance of non-adults | Average hourly distance by season per year | Season, Year, (Individual): Normal | |
