# Supplementary material for: Differential Range Use between Age Classes of Southern African Bearded Vultures Gypaetus barbatus
Source: PLoS One. 2014 Dec 31;9(12):e114920. doi: 10.1371/journal.pone.0114920 (PMC4281122; doi:10.1371/journal.pone.0114920)
Supplement: S2 Table — Details of the 18 Bearded Vultures caught in southern Africa and the tracking information used for analyses between September 2007 and April 2014. (DOCX) [file pone.0114920.s002.docx]

**Supporting Information**

**Table S2.** Details of the 18 Bearded Vultures caught in southern Africa and the tracking information used for analyses between September 2007 and April 2014.

| Age class | Individual | Sex | Transmission date († end date as a result of death of bird) | | Number of tracking months | Number of GPS fixes |
| --- | --- | --- | --- | --- | --- | --- |
|  |  |  | Start | End |  |  |
| Juvenile | 49182 | Female | 2007/09/06 | 2008/05/27† | 8 | 1 724 |
|  | 19853 | Male | 2009/08/08 | 2010/08/31 | 12 | 3 539 |
|  | 93462 | Male | 2009/08/20 | 2010/08/31 | 12 | 4 061 |
|  | 93463 | Female | 2009/08/26 | 2010/08/31 | 12 | 4 879 |
|  | 93465 | Female | 2010/07/06 | 2011/08/31 | 13 | 4 537 |
|  | 93468 | Female | 2009/08/12 | 2010/08/31 | 12 | 4 200 |
|  | 108925 | Female | 2012/07/29 | 2012/12/31 | 4 | 2 042 |
|  | 108926 | Male | 2012/09/02 | 2012/12/31 | 4 | 1 592 |
|  | 108927 | Male | 2012/09/03 | 2012/12/31 | 4 | 1 651 |
|  | 108928 | Male | 2012/09/17 | 2012/12/31 | 4 | 1 223 |
| Immature | 19853 | Male | 2010/09/01 | 2012/08/31 | 24 | 6 351 |
|  | 93462 | Male | 2010/09/01 | 2012/08/31 | 24 | 8 052 |
|  | 93463 | Female | 2010/09/01 | 2012/08/31 | 24 | 8 461 |
|  | 93465 | Female | 2011/09/01 | 2012/10/15† | 13 | 4 543 |
|  | 93468 | Female | 2010/09/01 | 2012/04/01† | 19 | 6 473 |
|  | 93461(1) | Female | 2009/08/23 | 2010/05/08 | 8 | 2 920 |
|  | 93464(1) | Male | 2010/07/06 | 2010/08/30† | 1 | 340 |
| Sub-adult | 19853 | Male | 2012/09/01 | 2013/03/31 | 7 | 1 825 |
|  | 93462 | Male | 2012/09/01 | 2013/08/31 | 12 | 3 446 |
|  | 93463 | Female | 2012/09/01 | 2013/08/31 | 12 | 3 880 |
| Adult | 93461(2) | Female | 2010/08/10 | 2012/12/31 | 28 | 16 420 |
|  | 93464(2) | Female | 2011/09/11 | 2014/04/30 | 31 | 9 651 |
|  | 93467 | Female | 2010/07/21 | 2012/08/10† | 24 | 12 821 |
|  | 93466 | Female | 2010/09/01 | 2014/04/30 | 44 | 18 185 |
|  | 108923 | Male | 2012/08/16 | 2013/12/31† | 16 | 7 356 |
|  | 108924 | Male | 2012/08/26 | 2014/04/30 | 20 | 6 435 |
| **Total** | **26** | **14F, 12M** | **-** | **-** | **392** | **146 607** |
