# Supplementary material for: Differential Range Use between Age Classes of Southern African Bearded Vultures Gypaetus barbatus
Source: PLoS One. 2014 Dec 31;9(12):e114920. doi: 10.1371/journal.pone.0114920 (PMC4281122; doi:10.1371/journal.pone.0114920)
Supplement: S3 Table — Bearded Vulture ranging information showing average hourly flight distances and kernel and MCP home range sizes in km2 depicted by individual separated by age class. (DOCX) [file pone.0114920.s003.docx]

**Supporting Information**

| Age | Individual | Sex | Average hourly flight distance (mean ± standard deviation) | Kernel home range | | | MCP  home range |
| --- | --- | --- | --- | --- | --- | --- | --- |
|  |  |  |  | 90% | 75% | 50% |  |
| Juvenile | 49182 | female | 6.5 ± 1.8 (0-43.1) | 27 288 | 14 483 | 6 006 | 33 504 |
|  | 19853 | male | 5.6 ± 0.9 (0-42.2) | 9 021 | 5 500 | 2 759 | 17 564 |
|  | 93462 | male | 5.9 ± 0.8 (0-42.8) | 15 386 | 8 700 | 3 814 | 31 205 |
|  | 93463 | female | 4.3 ± 0.9 (0-41.6) | 6 142 | 3 222 | 1 420 | 18 529 |
|  | 93465 | female | 5.0 ± 0.9 (0-51.1) | 16 739 | 9 611 | 4 015 | 32 606 |
|  | 93468 | female | 5.3 ± 1.1 (0-47.1) | 9 222 | 5 497 | 2 514 | 16 166 |
|  | 108925 | female | 4.8 ± 0.5 (0-38.5) | 6 862 | 3 886 | 1 687 | 13 654 |
|  | 108926 | male | 4.9 ± 0.6 (0-40.4) | 6 895 | 3 755 | 1 766 | 17 462 |
|  | 108927 | male | 3.7 ± 0.7 (0-47.1) | 3 515 | 2 176 | 1 121 | 5 565 |
|  | 108928 | male | 4.3 ± 0.9 (0-38.7) | 4 334 | 2 704 | 1 394 | 7 755 |
| Immature | 19853 | male | 6.3 ± 1.4 (0-52.1) | 25 145 | 14 575 | 7 028 | 48 013 |
|  | 93461(1) | female | 9.4 ± 1.7 (0-52) | 36 204 | 23 560 | 11 660 | 47 818 |
|  | 93462 | male | 6.9 ± 1.2 (0-60.5) | 23 793 | 13 875 | 6 166 | 46 278 |
|  | 93463 | female | 6.6 ± 1.7 (0-123) | 10 789 | 6 362 | 2 542 | 26 368 |
|  | 93464(1) | male | 8.5 (0-42.8) | 18 808 | 11 085 | 5 090 | 20 263 |
|  | 93465 | female | 7.2 ± 1.0 (0-61.7) | 23 449 | 13 613 | 6 689 | 35 546 |
|  | 93468 | female | 6.2 ± 1.4 (0-85.4) | 14 971 | 8 246 | 3 210 | 34 570 |
| Sub-adult | 19853 | male | 7.8 ± 1.8 (0-49.2) | 22 690 | 12 880 | 6 086 | 34 741 |
|  | 93462 | male | 8.5 ± 1.9 (0-109) | 34 109 | 20 329 | 10 088 | 51 620 |
|  | 93463 | female | 7.0 ± 1.6 (0-56.8) | 21 157 | 13 319 | 6 785 | 36 521 |
| Adult | 93461(2) | female | 4.4 ± 1.8 (0-62.8) | 127 | 70 | 29 | 12 343 |
|  | 93464(2) | female | 4.5 ± 1.4 (0-81.5) | 99 | 45 | 15 | 1 948 |
|  | 93466 | female | 3.3 ± 0.9 (0- 24.3) | 77 | 31 | 11 | 5 680 |
|  | 93467 | female | 3.8 ± 0.9 (0-56.7) | 315 | 136 | 63 | 27 245 |
|  | 108923 | male | 3.7 ± 5.3 (0-89.9) | 95 | 39 | 14 | 2 798 |
|  | 108924 | male | 4.0 ± 0.8 (0-183.8) | 1 000 | 362 | 149 | 62 494 |

**Table S3.** Bearded Vulture ranging information showing average hourly flight distances and kernel and MCP home range sizes in km^2^ depicted by individual separated by age class.
