# Supplementary material for: Differential Range Use between Age Classes of Southern African Bearded Vultures Gypaetus barbatus
Source: PLoS One. 2014 Dec 31;9(12):e114920. doi: 10.1371/journal.pone.0114920 (PMC4281122; doi:10.1371/journal.pone.0114920)
Supplement: S1 Form — Data Access Form. The satellite tracking data used in this study is available on request from Ezemvelo KwaZulu-Natal Wildlife. (DOC) [file pone.0114920.s004.doc]

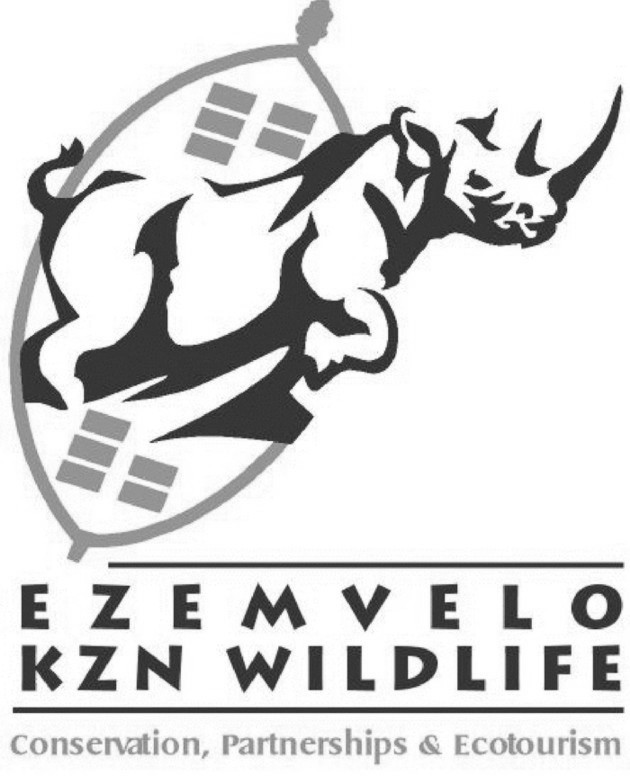


**BIODIVERSITY DATA REQUEST FORM**

| SUBMIT TO: |  | FROM: Date: |
| --- | --- | --- |
| Biodiversity Data Manager  Ezemvelo KZN Wildlife  P.O. Box 13053  CASCADES  3202 |  | Name & Address: |
| Email: database@kznwildlife.com |  | Email: |
| Fax: 033 - 845 1226 |  | Phone: |
|  |  | Fax: |

It is requested that the following data set be provided:

**General Description***

**Geographical and temporal extent of data** (area of coverage or bounding coordinates; time period (from – to))

**Specifications*** (e.g. software format)

:

**Purpose** * (what will the data be used for, nature of final product)

**Required date for delivery of data**:

* Adequate information given here will prevent unnecessary delays due to EKZNW having to call for further information.

**Conditions**

Recipients will need to undertake:

1. to use the data set only in accordance with the conditions specified;
2. to acknowledge the source of the data set as Ezemvelo KZN Wildlife (EKZNW) in any documentation derived from or associated with the use of these data;
3. to provide EKZNW with a free copy of any products generated in whole or in part from the data provided by EKZNW;
4. not to hold EKZNW liable for the quality or accuracy of the data supplied and to indemnify and hold harmless EKZNW, its members, officers, employees and agents against all claims, losses, legal proceedings or costs arising for whatever reason from use or dissemination of the data supplied;
5. not to distribute data obtained to third parties; and
6. to adhere to any further conditions that may be applied.……………………………………………
7. ……………………………………………………………………………………………………………..………………………………………………………………………………………………………..……

**Costs** (will be waived for registered projects)

1. Retrieval of information from databases - R50 per 15 minutes or part thereof.
2. Work involving professional services other than retrieval from EKZNW databases will be negotiated.

By signing this form the signatory accepts the conditions and costs as stated above.

Name.........................................................................................ID Number:……………..

Signature:…………………..
